# Overexpression of CYP11A1 recovers cell cycle distribution in renal cell carcinoma Caki-1

**DOI:** 10.1186/s12935-022-02726-4

**Published:** 2022-10-01

**Authors:** Hien Thi My Ong, Tae-Hun Kim, Eda Ates, Jae-Chul Pyun, Min-Jung Kang

**Affiliations:** 1 Center for Advanced Biomolecular Recognition, Korea Institute of Science and Technology, Seoul, 02792 Republic of Korea; 2 Division of Bio-Medical Science & Technology, KIST School, University of Science and Technology, Seoul, 02792 Republic of Korea; 3 Department of Materials Science and Engineering, Yonsei University, Seoul, 03722 Republic of Korea

**Keywords:** CYP11A1 overexpression, Cell cycle, G2/M arrest, Clear cell renal carcinoma suppression

## Abstract

**Background:**

Clear cell renal carcinoma is commonly known for its metastasis propensity to outspread to other organs and is asymptomatic in the early stage. Recent studies have shown that deficiencies in CYP11A1 expression can lead to fatal adrenal failure if left untreated and are associated with downstream regulation in various cancer types. However, the molecular mechanisms of CYP11A1 and kidney cancer proliferation remain unclear.

**Methods:**

Normal and renal carcinoma cell lines (HEK293 and Caki-1) were transfected with plasmid encoding CYP11A1 to overexpress the P450scc protein. Cell cycle distribution was investigated using flow cytometry. The expression of proteins related to C-Raf/ERK/JNK/p38 signaling pathways was examined using western blot.

**Results:**

We observed that CYP11A1 overexpression suppressed the cyclin B1 and cell-division cycle 2 expression while cyclin-dependent kinases 2 and 4 were unaffected. Cancer cell migration and invasion were suppressed along with epithelial-intermediate metastatic markers Snail and Vimentin. In addition, in CYP11A1-overexpressing Caki-1 cells, cdc2/cyclinB1 was downregulated while the phosphorylation of cdc25c, a G2/M arrest-related upstream signal, was increased. The intrinsic-mitochondrial apoptosis markers were not significantly altered. We also identified that the C-Raf/ERK/JNK/p38 pathway is an important pro-apoptotic mechanism in CYP11A1-overexpressing cell-based models. Our results suggest that CYP11A1 overexpression recovered the disturbed cell cycle arrest distribution in renal carcinoma cell line Caki-1 through G2/M arrest and C-Raf/ERK/JNK pathway.

**Conclusions:**

Our findings may suggest promising new therapeutic targets to suppress kidney cancer proliferation without affecting normal cells, eventually improving the survival of patients with cancer.

**Supplementary Information:**

The online version contains supplementary material available at 10.1186/s12935-022-02726-4.

## Background

Renal cell carcinoma (RCC) is a heterogeneous cancer group including clear cell RCC, papillary RCC, and chromophore RCC in which clear cell RCC occurs in renal tubular epithelial cells of the kidneys and is the most common subtype [1]. The incidence of RCC varies greatly around the world, with approximately 403,000 cases and 175,000 kidney cancer-related deaths in 2018 [2]. Early diagnosis of local diseases results in a survival rate of 70–94% [3]. Recent years have witnessed a rise in major practice-changing trials for the treatment of RCC as the metastatic stage that completely converted the therapeutic goal for this disease. The vascular endothelial growth factor (VEGF) pathway has been investigated as a key mediator in RCC development [4]. Specific inhibitors that block immune responses, such as nivolumab and ipilimumab, are now approved for primary treatment of patients with metastatic RCC [5]. Results from various clinical trials demonstrated valuable advantages of a combination of VEGF therapy and immune response inhibition, renewing hope for treating most at-risk RCC [6]. The role of surgery for metastatic RCC has been defined, and the initial tumor resection did show clinical benefits. However, even if detected early, patients with RCC almost certainly cannot undergo curative nephrectomy [7]. Furthermore, metastases often cause death in these patients due to the long-term risk of local or systemic recurrence to the opposite kidney. Abnormal lipid metabolism was previously proposed as the clinical outcome of metastatic RCC. Pregnenolone is an upstream steroid that acts as a precursor or metabolic component in the biosynthesis of other downstream steroid hormones, including progestogens, estrogens, androgens, glucocorticoids, and mineralocorticoids [8]. Glucocorticoids are frequently applied as a therapeutic or clinical treatment for various cancers, including RCC [9]. Glucocorticoids also reportedly weaken mobility and invasiveness while eliciting a mesenchymal to epithelial-like transition of cancer cells [10]. Nevertheless, present target therapies are less effective against late-stage RCC and can cause drug resistance or unwanted side effects.

Cytochrome P450s (CYPs) are critical regulators of pharmacokinetics and drug metabolism. Previous studies demonstrated that the CYP11 family is involved in steroid biosynthesis, while CYP11A1 plays a key role in the first-step cleavage of cholesterol to synthesize pregnenolone and other steroids. Notably, abnormal steroid production is associated with numerous cancer grades and conditions. Recent studies have shown that CYP11A1 is downregulated in various cancer types [11]. In this regard, it is vital to highlight the molecular mechanisms of CYP11A1 and kidney cancer and identify novel, potentially effective therapeutics. Targeting CYP11A1 might offer a new perspective on cancer prevention and treatment. Our findings suggest that CYP11A1 may act as a therapeutic target to suppress kidney cancer proliferation while causing negligible effects on normal cells.

## Materials and methods

### Cell culture and protein quantification

Human normal epithelial kidney (HEK293) and renal cancer (Caki-1) cell lines were purchased from the Korea Cell Line Bank (KCLB) and cultured in Dulbecco’s modified Eagle medium (DMEM)-medium–high glucose (GenDEPOT, TX, USA) containing 10% fetal bovine serum (GIBCO, MA, USA) and 1% penicillin/streptomycin (GIBCO, MA, USA). Culture plates were maintained at 37 °C with 5% CO_2_ in a humidified incubator. For protein quantification, cultured cells were rinsed twice with ice-cold phosphate-buffered saline (PBS) and lysed using Pierce^™^ RIPA Lysis Buffer (Thermo Fisher Scientific, MA, USA) accompanied with Protease/phosphatase Inhibitor Cocktail (Cell Signaling Technology, MA, USA). Cell lysates were incubated at 4 °C for 20 min and centrifuged at 10,000×*g* for 20 min at 4 °C. The supernatant was collected, and Pierce^™^ BCA Protein Assay Kit (Thermo Fisher Scientific, MA, USA) was used to measure the protein concentrations according to the manufacturer’s protocol.

### Plasmid DNA purification and transfection

CYP11A1 cDNA with a pCMV-SPORT5 vector (Clone ID: hMU004796) was used for cell transfection. The clone was provided by Korea Human Gene Bank, Medical Genomics Research Center, KRIBB, Korea. Luria–Bertani broth media (25 g/L) was prepared by adding an antibiotic including 100 μg/mL ampicillin. According to the manufacturer’s instructions, plasmid DNA was isolated using Plasmid Midi Kit (Qiagen, MD, USA) after culturing competent cells overnight. CYP11A1 cDNA concentration was measured by Nano-drop (Thermo Fisher Scientific, MA, USA). Lipofectamine 3000 Transfection Reagent (Invitrogen, MA, USA) was used to perform transient transfection, following Invitrogen’s protocol. The transfection efficiency of CYP11A1 was confirmed by western blotting; an increasing amount of plasmid DNA (1000 ng, 2000 ng, and 4000 ng) was tested for the optimal concentration.

Cells were seeded onto glass coverslips and transfected with CYP11A1, similar to the procedure above. Next, cells were fixed in 4% formaldehyde (Sigma-Aldrich, MA, USA) for 15 min, and immunofluorescence staining was performed per the manufacturer’s protocol. After blocking the nonspecific binding, cells were incubated with the primary antibody CYP11A1 at 4 °C overnight, thoroughly washed with 1×PBS, then incubated with the secondary antibody Alexa Fluor^®^ 488 Conjugate Anti-Rabbit IgG (H + L; Cell Signaling Technology, MA, USA) for 2 h incubation at room temperature. The nuclei were stained with Hoechst 33,342 (Thermo Fisher Scientific, MA, USA) for 10 min, coverslips were mounted with mounting solution, and images were acquired with EVOS^™^ M7000 Imaging System (Thermo Fisher Scientific, MA, USA).

### Wound healing, invasion, and reactive oxygen species assay

A wound healing assay was performed 24 h after transfection. Uniform wounds were created across the confluent cell monolayer using a sterilized 200 μL pipette tip. Cell debris was removed by repeated washing with 1×PBS, and cells were allowed to migrate into the wound area for 24 h at 37 ℃. Digital photos were taken at 0 and 24 h after the scratch. ImageJ 1.53a (National Institutes of Health, Bethesda, MD, USA) was used to calculate the wound width at four locations within each well. The percentage of wound closure was quantified by dividing the healed wound width at 24 h by the initial width. Each experiment was performed three times using triplicate wells.

Matrigel-invasion assay was performed using Transparent PET Membrane chambers with an 8.0 µm pore size. Cells (1 × 10^5^ cells/well) were resuspended in 100 µL DMEM serum-free media and added to the upper chamber, coated with Matrigel (BD Biosciences, NJ, USA). The lower cavity of the transwell was filled with 500 µL 5% FBS medium containing fibronectin (5 µg/mL) as a chemoattractant. After 24 h, cells were fixed with 4% formaldehyde and permeabilized with 100% methanol, followed by staining with Giemsa (Merck, NJ, USA) for 15 min at room temperature. The upper chamber was cleaned with a cotton swab. Cells were counted under a fluorescence microscope (Nikon Eclipse TE 2000-U, NY, USA) by randomly selecting multiple fields per membrane. The invasiveness of cells was expressed as the mean number of cells that invaded the lower chamber. Each experiment was performed in triplicate.

EZ-Hydrogen Peroxide/Peroxidase assay kit (DooGen Bio, Seoul, Korea) was used to measure oxidative stress. Cell culture supernatant was centrifuged at 10,000×*g* for 5 min to remove insoluble particles. A standard curve was generated using the same non-conditioned media according to the manufacturer’s instructions. Absorbance at 560 nm was measured using a spectrophotometer (Bio-Rad, CA, USA).

### Flow cytometry analysis of cell cycle and apoptosis

Cultured cells (3 × 10^6^ cells/mL) were harvested and washed with PBS before exposure to staining buffer. Annexin V and PI staining were performed using the Alexa Fluor^®^ 488 Annexin V/Dead Cell Apoptosis Kit (Invitrogen, MA, USA), according to the manufacturer’s instructions. For cell cycle analysis, harvested cells were fixed with 70% ethanol, washed twice with cold PBS, and centrifuged to discard the supernatant. The pellet was re-suspended in PI staining buffer containing a mixture of 50 µg/mL PI and RNase A/T1 (0.5 µg/mL) and incubated at 4 ℃ overnight in the dark. Data were analyzed using a FACS Calibur instrument and Cell Quest software (BD Biosciences, NJ, USA). Flow cytometry analysis was performed at excitation wavelengths of 488 nm and 535 nm to detect apoptosis; 5000 HEK293 and Caki-1 cells were used to analyze apoptosis. DNA content and apoptosis of HEK293 and Caki-1 cells were analyzed using FlowJo Software (Tree Star Inc., Ashland, OR, USA). Threshold triggers were set on forward and side scatters to eliminate background noise and specifically analyze cells. The fluorescence signal was calculated as the mean intensity in arbitrary units (au).

### Western blot analysis

Protein samples were separated by one-dimensional 12% Tris–glycine SDS-PAGE, then transferred to nitrocellulose membranes (Bio-Rad, CA, USA). For membrane blocking, membranes were incubated with 1×TBST containing 5% skim milk for 1 h after washing with 1×TBST. Nitrocellulose membranes were incubated overnight with the primary antibodies 1:1000 in 5% BSA at 4 ℃ in the dark. After washing three times with TBST for 5 min, 1:5000 horseradish peroxidase-conjugated secondary antibodies (Cell Signaling Technology, MA, USA) were added to 5% skim milk, and membranes were incubated at room temperature for 1 h. Blots were detected using a chemiluminescence SignalFire^™^ ECL Reagent (Cell Signaling Technology, MA, USA) by Ez-Capture MG system (ATTO, NY, USA), and the relative intensity of the western blot bands was measured using ImageJ 1.53a (National Institutes of Health). GAPDH antibody, purchased from GeneTex (CA, USA), was used as a loading control. The antibodies used in this study included anti-CYP11A1, anti-Snail, anti-Vimentin, anti-Cdk2, anti-Cdk4, anti-phospho-p44/42 MAPK (Erk1/2) (Thr202/Tyr204), anti-pp44/42 MAPK (Erk1/2), anti-cdc25c, and anti-phospho-cdc25c Ser216. Cell Cycle/Checkpoint Antibody Sampler Kit, Apoptosis Antibody Sampler Kit, and Pro-Apoptosis Bcl-2 Family Antibody Sampler Kit were also purchased from Cell Signaling Technology (MA, USA).

### Statistical analysis

All measures were replicated at least three times, and the corresponding data are presented as the mean ± SD. All data were analyzed with Origin 2022 (OriginLab, MA, USA). One-way ANOVA and Student’s t-test were performed for intergroup comparisons. Western blot results were reported as fold changes compared to control samples, and GAPDH was used as the loading control. Differences were considered statistically significant at *P ≤ 0.05, **P ≤ 0.01, and ***P ≤ 0.005.

## Results

### CYP11A1 overexpression inhibits the EMT process and cancer cell mobility

**Fig. 1:**
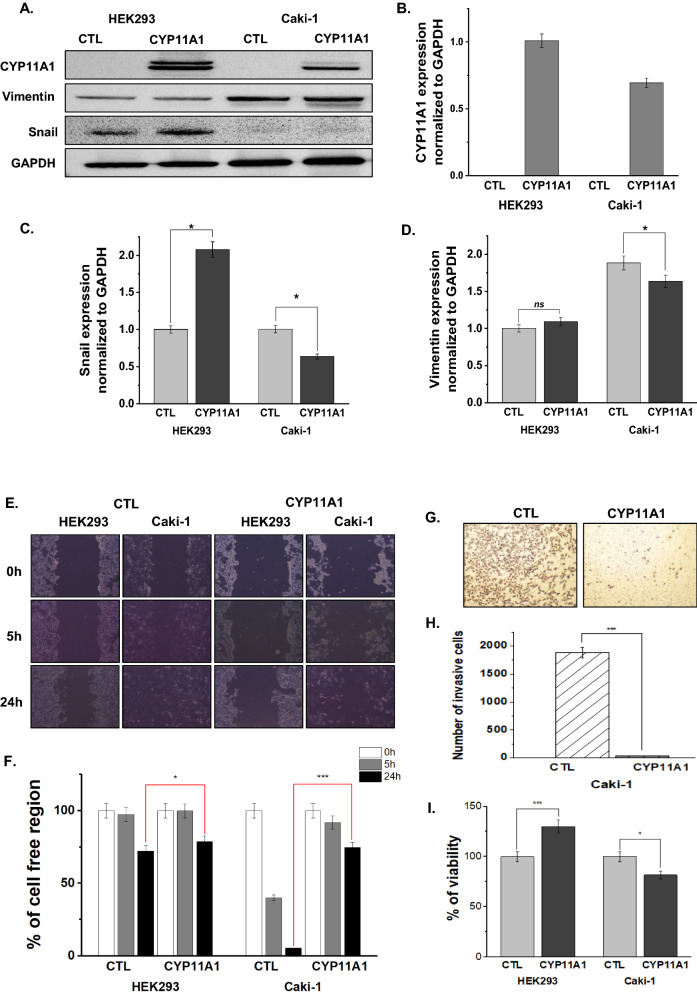
Effects of CYP11A1 overexpression on cancer (Caki-1) and normal kidney (HEK293) cell lines. **A** Western blot of CYP11A1, Vimentin, and Snail. **B, C, D** Protein expression levels normalized to GAPDH. Inhibition of cell migration (**E, F**), invasion (**G, H**), and cell viability (**I**) after CYP11A1 transfection

HEK293 cell line is a normal kidney epithelial cell model used as control, while the Caki-1 cell line represents an in vitro model system for a specific type of kidney cancer that exhibits extensive vasculature and standard features of RCC. To investigate the effects of CYP11A1 overexpression on kidney cancer cells, cell proliferation, invasion, and viability were measured. Western blotting (Fig. 1A, B) and immunofluorescence (Additional file 1: Fig. S1) were used to confirm the overexpression of CYP11A1 and EMT protein markers. Our results revealed a significant reduction in Vimentin (Fig. 1C) and Snail (Fig. 1D) expression in Caki-1 cells after CYP11A1 transfection, while normal kidney HEK293 cells showed a reversible increase in protein levels. A rapid decrease in mesenchymal protein levels in the cancer cell line indicates that CYP11A1 overexpression is associated with inhibiting cancer cell mobility. This in vitro transfection system has been used for the molecular mechanism study of CYP11A1 effects on the inhibition of cancer cell mobility and proliferation. Migration distances were monitored at 0, 5, and 24 h (Fig. 1E). The wound healing assay showed no significant effect of CYP11A1 transfection in HEK293 cells. However, non-transfected Caki-1 cells migrated, covering approximately 90% of the wound region. The wound width was approximately 75% of the initial width in CYP11A1-transfected Caki-1 cells (Fig. 1F), indicating that CYP11A1 overexpression highly inhibited cell migration, specifically in cancer cells. The matrix-gel invasion assay showed that Caki-1 cell invasion was significantly reduced from 1.87 × 10^3^ cells/field in the control group to 0.03 × 10^3^ cells/field after CYP11A1 overexpression (Fig. 1G). The number of invasive CYP11A1-overexpressing Caki-1 cells rapidly decreased compared to the non-transfected control group (Fig. 1H). Cytotoxicity was measured to determine the inhibitory effect of CYP11A1 in cancer cells. Our results showed that CYP11A1 did not inhibit HEK293 cell proliferation, while a significant reduction in Caki-1 cell viability was evident 24 h after transfection (Fig. 1I). Together with the migration, invasion, and viability assay findings, these results confirm that CYP11A1 overexpression inhibits cancer cell proliferation in kidney cancer cells.

### CYP11A1 overexpression recovers cell cycle distribution in cancer cell line

**Fig. 2:**
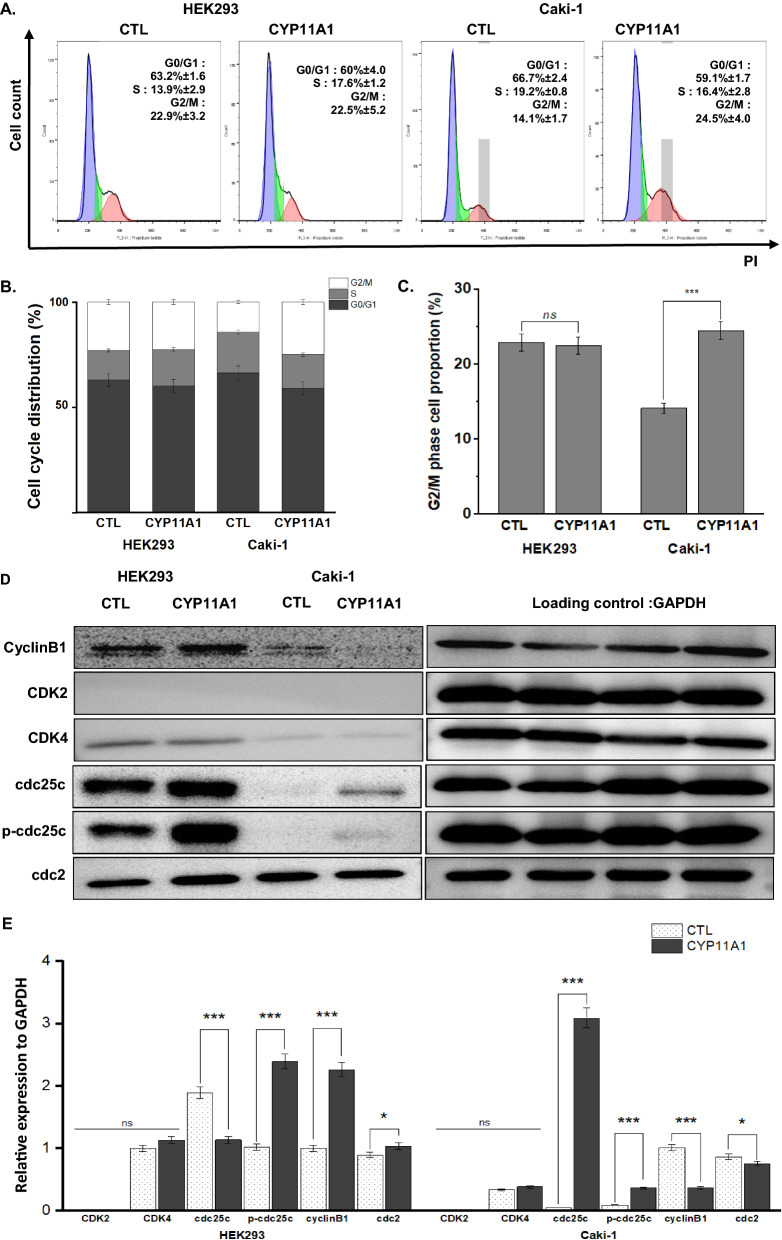
G2/M phase arrest by CYP11A1-overexpressing cancer cells. **A** DNA content of PI-stained cells detected using flow cytometry. **B** The percentage of cells in each phase and **C** the total cell proportion in the G2/M phase. **D** Western blotting of CDK2, CDK4, cdc25c, p-cdc25c, cyclinB1, and cdc2 expression. **E** The expression levels of these cyclin-dependent kinases were normalized to GAPDH

To explore the effects of CYP11A1 overexpression on cancer cell mobility and proliferation, cell cycle distribution profiles were analyzed using flow cytometry (Fig. 2A, B). In the Caki-1 group, the majority of cells were increased at the G2/M phase after 24-h CYP11A1 transfection, while the percentage of HEK293 cells in all phases displayed no significant difference (Fig. 2C). These results indicated that CYP11A1 overexpression arrested the cancer cell cycle at G2/M phase, resulting in a similar cell cycle distribution to that in control HEK293 cells. The distribution of non-transfected Caki-1 cells was reduced compared with that of normal kidney epithelial cells and was recovered by CYP11A1 transfection.

During cell cycle progression, the G2/M phase is triggered by the activation of the cyclin B1/Cdc2 kinase complex, which is controlled by various phosphorylation-dephosphorylation factors. To study the mechanism underlying the CYP11A1 overexpression-mediated induction of G2/M phase arrest, the cyclin-dependent kinases (CDK) family and cell cycle regulatory proteins were examined using western blotting (Fig. 2D, E). The results showed that CDK2 and CDK4 expressions were not significantly changed in either group. In CYP11A1-overexpressing HEK293 cells, cdc25C expression was decreased, resulting in p-cdc25c upregulation, accompanied by an increased amount of detected cylinB1/cdc2. However, cyclin B1 and cdc2 expression levels were downregulated in Caki-1 cells after CYP11A1 transfection, indicating that the cyclin B1/Cdc2 complex could be suppressed, and the cell cycle was arrested in the G2/M phase. All these observations confirmed that CYP11A1 overexpression induces G2/M phase arrest in kidney cancer cells by regulating cell cycle-related kinases. Interestingly, the cell cycle distribution of Caki-1 cells was restored to a normal state as in normal kidney epithelial cells (HEK293).

### CYP11A1 promoted ROS and activated apoptosis

**Fig. 3:**
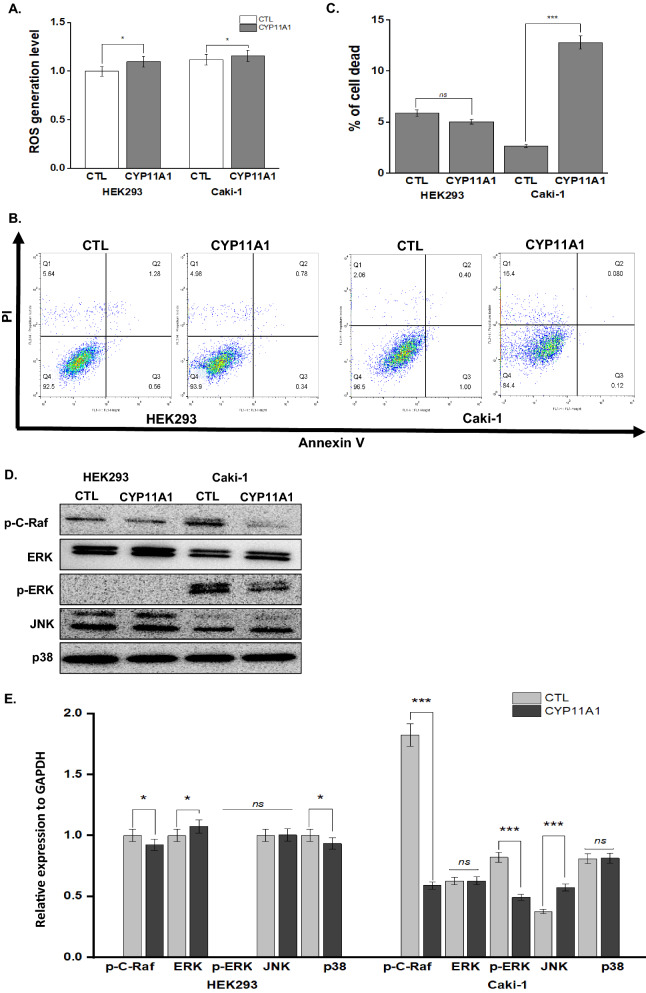
CYP11A1 promotes apoptosis by inhibition of MAPK signaling. **A** ROS levels determined by the concentration of hydrogen peroxide generated under oxidative stress conditions. **B** Apoptosis of HEK293 and Caki-1 cells transfected with CYP11A1 for 24 h detected using Annexin V-PI double staining followed by flow cytometry. **C** The percentage of dead cells in each group. **D** Western blotting of p–C-Raf, ERK, p-ERK, JNK, and p38 expression. **E** protein expression levels were normalized to GAPDH

Our study demonstrated a slight rise in ROS levels by CYP11A1 overexpression in both HEK293 (fold change 1.11, p < 0.05) and Caki-1 (fold change 1.03, p < 0.05) cell lines (Fig. 3A). Following transfection with CYP11A1 for 24 h, the apoptotic rate was assessed by FACS using Annexin V/PI double staining (Fig. 3B). The results revealed that CYP11A1 overexpression significantly increased the apoptotic rate of Caki-1 cells from 2.66 to 12.8%, while the apoptotic rate of control HEK293 cells was 5.91% and 5.05% in CYP11A1-overexpressing HEK293 cells (Fig. 3C). To identify the mechanism underlying CYP11A1-induced apoptosis, we performed western blot analysis of apoptosis-associated proteins 24 h after transfection. The activity of caspase 3 and 7 remained stable, whereas caspase 9 and PARP were downregulated in Caki-1 cells (Additional file 1: Fig. S2). However, the expression of cleaved caspase 3, 7, and 9, as well as PARP and Bcl-2 families, could not be determined in any of the groups. (Additional file 2) These results confirmed that the association between CYP11A1 and apoptosis was not subjected to caspase-related death receptor (extrinsic) and mitochondria-dependent (intrinsic) pathways. We next explored the MAPK pathway that plays a key role in regulating proliferation, differentiation, and survival of CYP11A1-driven ROS in apoptosis. The phosphorylation levels of MAPK family members, including p-C-Raf, p-ERK, ERK, JNK, and p38, were examined between CYP11A1-transfected and non-transfected groups (Fig. 3D, E). CYP11A1-overexpressing Caki-1 cells showed sustained activation of JNK and p38 along with inhibition of p-ERK and p-C-Raf, supporting our hypothesis that CYP11A1-induced oxidative stress may activate JNK-p38 pathways, specifically promoting apoptosis in the cancer cell line. The inhibition of p-ERK and p-C-Raf may be related to reduced cell proliferation in kidney cancer cells.

## Discussion

Cytochrome P450 is known to execute a series of cellular oxidation reactions that facilitate oxidative transformation, contributing to the production of intracellular ROS. ROS generated by CYP11A1 in mitochondria could induce oxidative stress and promote cell death [12]. Decreased expression of CYP11A1 influences the biosynthesis of steroid hormones and is related to several cancers. Recent studies have investigated the role of steroid hormones in various cancer types such as breast [13, 14], prostate [15], lung [16], liver [17], and colon cancer [18]. However, the molecular mechanisms underlying the anticancer effects of CYP11A1 on RCC are largely unknown. CYP11A1 reportedly triggers excessive oxidative stress in mitochondria against a human placental cell line derived from choriocarcinoma [19].

Our results suggested that CYP11A1 can suppress the EMT process by downregulating Snail and Vimentin levels. This effect was more pronounced in Caki-1 cells, a model of RCC metastasis that represent the phenotypic and functional patterns of well-differentiated proximal tubule epithelium cells [20].

The transition from G2 to M phase was achieved by activating the cdc2/cyclin B complex via cdc25c phosphorylation-induced upregulation of cdc2, while increased cdc25c phosphorylation could lead to cell cycle arrest in the G2 phase [21]. Our study found that CYP11A1 induced a remarkable increase in p-cdc25c and a decrease in cyclin B1 and cdc2 in Caki-1 cells, while the HEK293 cell line exhibited reversible changes in the expression of these kinases. These results suggest that CYP11A1 is involved in the G2/M phase arrest by positively suppressing the cdc2/cyclin B complex pathway via cdc25c phosphorylation. The regulation of cell cycle distribution after CYP11A1 overexpression resulted in the recovery of a cell cycle distribution resembling that of normal kidney epithelial cells (HEK293). In RCC, transglutaminase 2 intersects with p53, leading to p53 depletion and apoptosis avoidance [22]. Although the loss of p53 function is frequently related to the induction of cell cycle arrest [23], we did not observe p53 expression in Caki-1 cells. Programmed cell death is mainly regulated by intrinsic, extrinsic, and MAPK pathways, which are activated by the disruption of intracellular homeostasis, DNA damage, and stress response. The Bcl-2 family includes various pro-apoptotic proteins such as Bax, Bad, and Bim, the main regulators of the intrinsic pathway. A previous study reported the aberrant expression of Bcl-2 according to cancer cell type, revealing that Bcl-2 was downregulated in most cancers [24]. Normally, the activation of Bax and Bak proteins can trigger cytochrome C release following the extrinsic activation of caspase-3, -7, -9, and PARP, thus leading to apoptosis [25]. Our study further observed a rapid decrease in caspase-9 and PARP expression despite the lack of any significant difference in caspase-3 and -7 after CYP11A1 transfection. These results revealed that CYP11A1-induced apoptosis was relatively independent of Bcl-2 and caspase family activities. Recent studies have shown that the cellular signal transduction pathway C-Raf/MEK/ERK can act on key effectors to regulate cell proliferation [26, 27]. ERK1/2 activation usually leads to cell proliferation, resulting in initial hallmarks of various cancer activities. Consequently, factors involved in the ERK pathway are considered prospective therapeutic targets for cancer. A large number of regulators and inhibitors of this pathway are already being used in clinical trials. Notably, ERK1/2 activation can cause cell death, resulting in pro-apoptotic functions [28]. We found that CYP11A1-overexpressing Caki-1 cells halted C-Raf/ERK pathway by downregulating C-Raf and ERK1/2 phosphorylation. In response to oxidative stress, MAPK family members are crucial contributors to apoptosis via various signaling pathways. For instance, upregulation of JNK and p38 expression was considered stress-responsive and promoted programmed cell death [29].

**Fig. 4:**
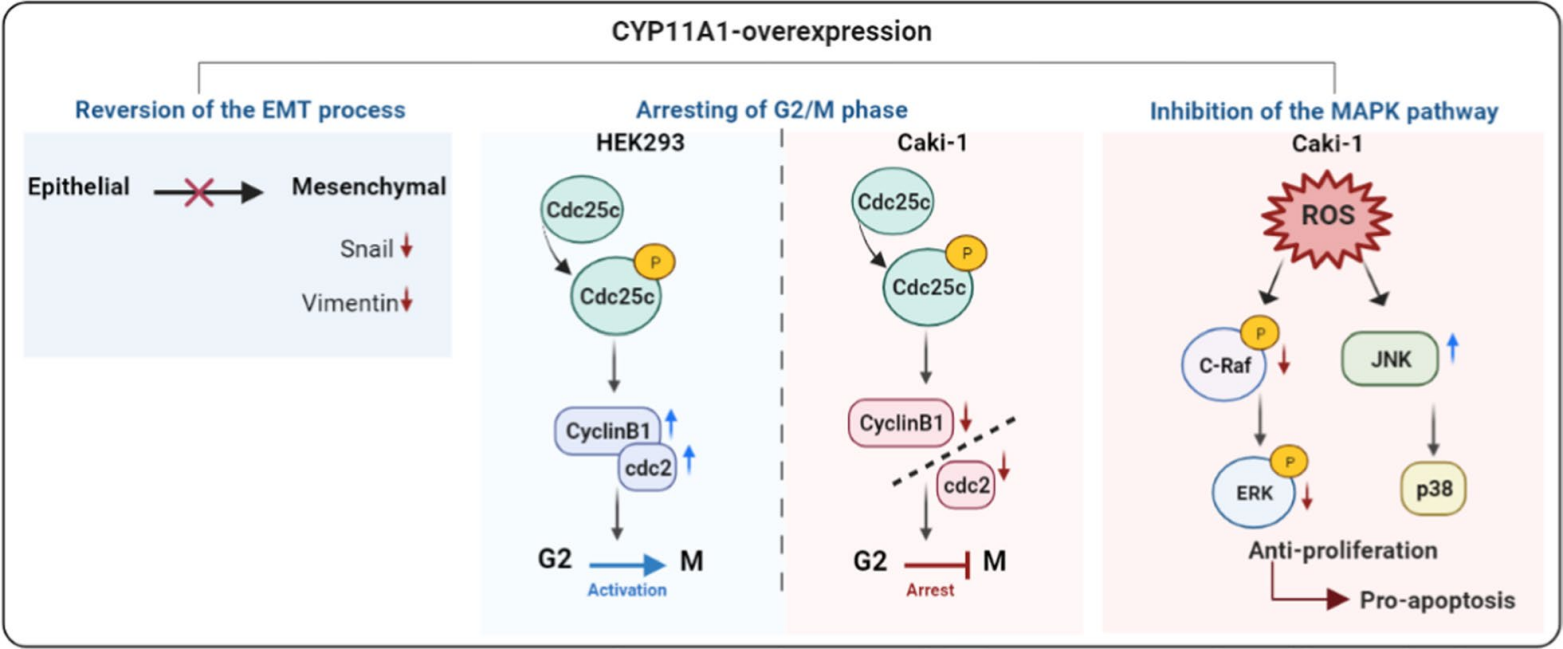
Schematic illustration displaying the mechanism of anticancer effect of CYP11A1: CYP11A1 reverses the EMT process, induces the G2/M phase arrest by suppression of cdc2/cyclinB1 complex, and inhibits the MAPK pathway by generating ROS

In conclusion, we demonstrated for the first time that CYP11A1 overexpression reversed the EMT process in Caki-1 cells, induced G2 phase arrest, generated ROS, and promoted apoptosis via the MAPK pathway (Fig. 4). All these findings suggest that CYP11A1 might exert anti-tumor effects on RCC. CYP11A1 could specifically target cancerous cells with minimal side effects on surrounding healthy cells. Cancer cell lines are widely used in cancer research as initial screening platforms for therapeutic targets. Nevertheless, in vitro systems cannot be fully controlled due to over-growth, nor can they simulate metastasis, which causes the invasion of surrounding tissues and other organs via blood vessels. Moreover, the molecular mechanism of CYP11A1 should be further explored in 3D in vitro tumor models. This study highlights that CYP11A1 could function as a potent anticancer target. Nevertheless, the key regulators underlying the CYP11A1-mediated suppression of cancer cell proliferation still warrant further investigation.

## Supplementary Information

**Additional file 1: Figure S1. **CYP11A1-overexpression in HEK293 (A) and Caki-1 (B) cells. CYP11A1 was performed with green color while blue color indicated nucleus. **Figure S2. **Western blotting performed to determine the expression level of caspase-3,7,9 and PARP.

**Additional file 2. **

## Data Availability

The dataset supporting the conclusions of this article is available from the corresponding author.

## Abbreviations

Bcl-2: B-cell lymphoma 2
BSA: Bovine serum albumin
Caspases: Cysteine–aspartic proteases
CDC2: Cell-division cycle 2
CDK2: Cyclin‐dependent kinase 2
CDK4: Cyclin‐dependent kinase 4
EMT: Epithelial to mesenchymal transition
ER: Endoplasmic reticulum
ERK: Extracellular signal-regulated kinases
JNK: C-Jun N-terminal kinases
MAPK: Mitogen-activated protein kinase
PBS: Phosphate-buffered saline
RCC: Renal cell carcinoma
TBS: Tris-buffered saline
TBST: TBS with 0.5% Tween 20
WST: Water-soluble tetrazolium salt
