# Supplementary material for: Overexpression of CYP11A1 recovers cell cycle distribution in renal cell carcinoma Caki-1
Source: Cancer Cell Int. 2022 Oct 1;22:299. doi: 10.1186/s12935-022-02726-4 (PMC9526279; doi:10.1186/s12935-022-02726-4)
Supplement: Supplementary file 1 — Additional file 1: Figure S1. CYP11A1-overexpression in HEK293 (A) and Caki-1 (B) cells. CYP11A1 was performed with green color while blue color indicated nucleus. Figure S2. Western blotting performed to determine the expression level of caspase-3,7,9 and PARP. [file 12935_2022_2726_MOESM1_ESM.docx]

**Supplementary data for:**

**Overexpression of CYP11A1 recovers cell cycle distribution in renal cell carcinoma Caki-1**

Hien Thi My Ong^1,2^, Tae-Hun Kim^3^, Eda Ates^1,2^, Jae-Chul Pyun^3^ and Min-Jung Kang^1,2*^

^1^Center for Advanced Biomolecular Recognition, Korea Institute of Science and Technology, Seoul, 02792 Republic of Korea.

^2^Division of Bio-Medical Science &Technology, KIST School, University of Science and Technology, Seoul, 02792 Republic of Korea.

^3^Department of Materials Science and Engineering, Yonsei University, Seoul, 03722 Republic of Korea

*** Corresponding author**

Dr. Min-Jung Kang

Center for Advanced Biomolecular Recognition

Korea Institute of Science and Technology

Seoul, 02792, Republic of Korea

Tel: +82-2-958-5088

E-mail address: [mjkang1@kist.re.kr](mailto:mjkang1@kist.re.kr)

**Fig S1.** CYP11A1-overexpression in HEK293 (A) and Caki-1 (B) cells. CYP11A1 was performed with green color while blue color indicated nucleus.


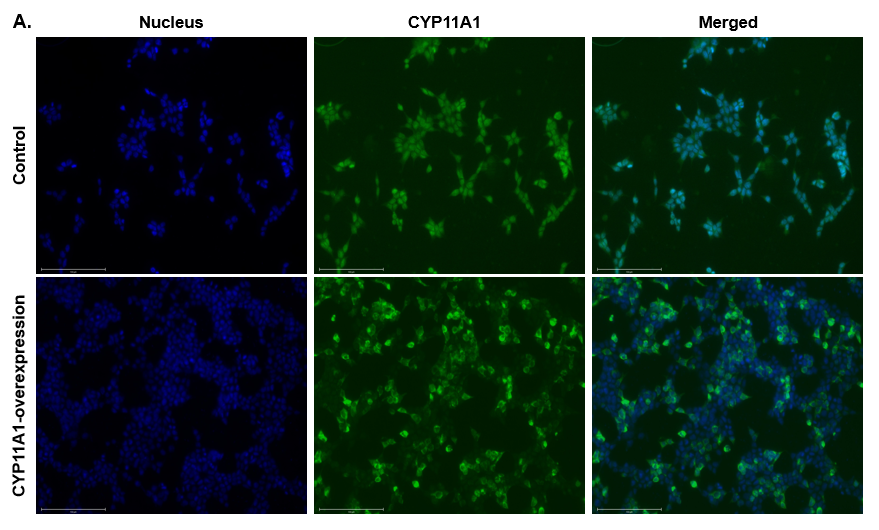


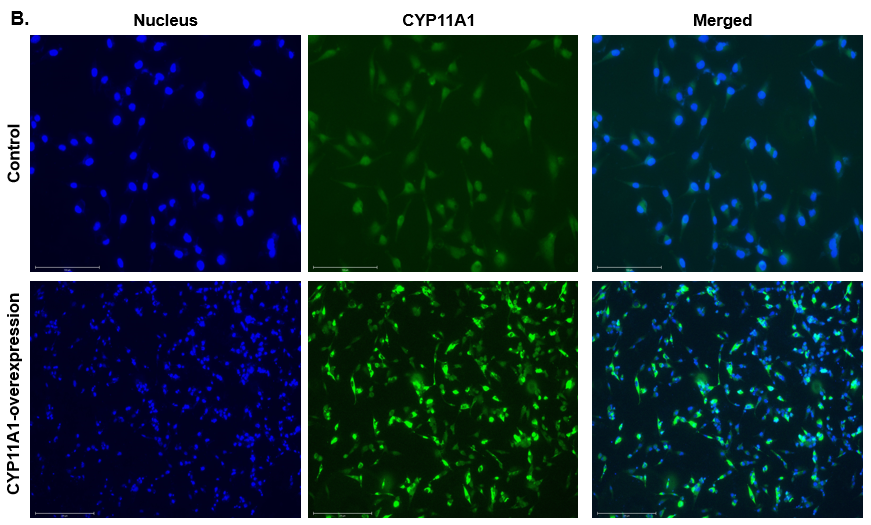


**Fig S2.** Western blotting performed to determine the expression level of caspase-3,7,9 and PARP.


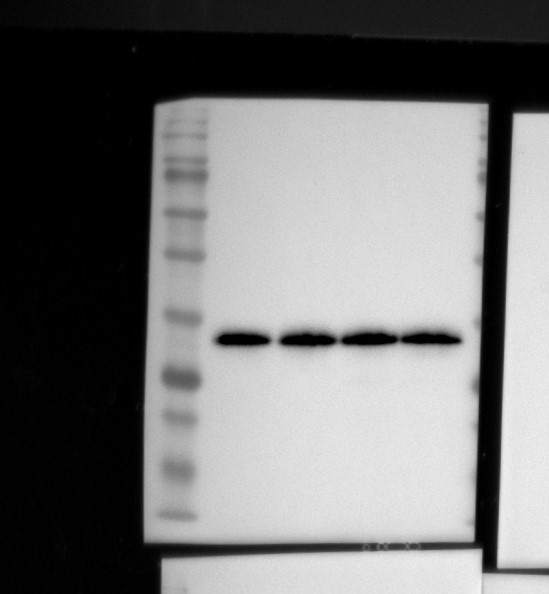

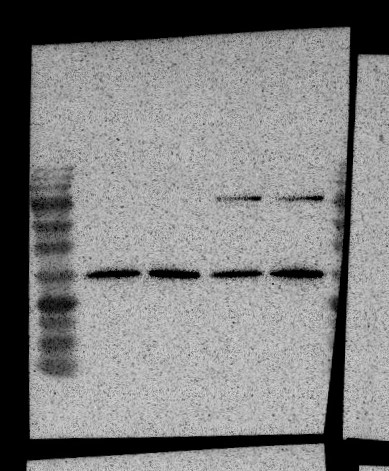

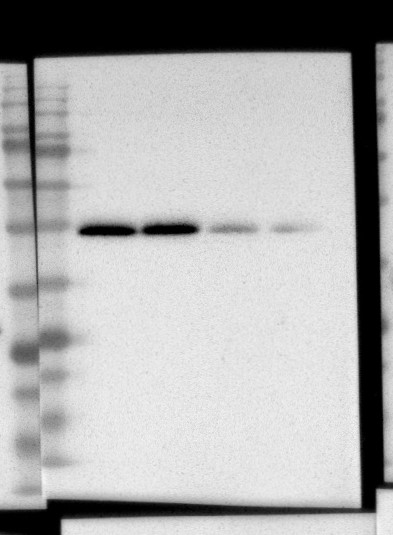

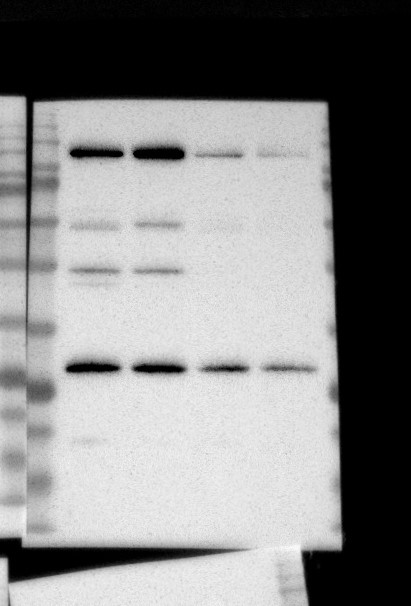

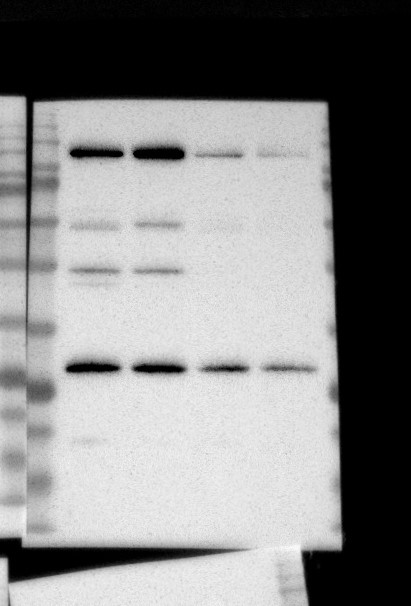


**caspase-3**

**caspase-7**

**caspase-9**

**24kDa PARP**

**116kDa PARP**

**CTL**

**CYP11A1**

**HEK293**

**CYP11A1**

**CTL**

**Caki-1**
