# Supplementary material for: Overexpression of CYP11A1 recovers cell cycle distribution in renal cell carcinoma Caki-1
Source: Cancer Cell Int. 2022 Oct 1;22:299. doi: 10.1186/s12935-022-02726-4 (PMC9526279; doi:10.1186/s12935-022-02726-4)
Supplement: Supplementary file 2 — Additional file 2. [file 12935_2022_2726_MOESM2_ESM.docx]

**Supplementary file for:**

**Overexpression of CYP11A1 recovers cell cycle distribution in renal cell carcinoma Caki-1**

Hien Thi My Ong^1,2^, Tae-Hun Kim^3^, Eda Ates^1,2^, Jae-Chul Pyun^3^ and Min-Jung Kang^1,2*^

^1^Center for Advanced Biomolecular Recognition, Korea Institute of Science and Technology, Seoul, 02792 Republic of Korea.

^2^Division of Bio-Medical Science &Technology, KIST School, University of Science and Technology, Seoul, 02792 Republic of Korea.

^3^Department of Materials Science and Engineering, Yonsei University, Seoul, 03722 Republic of Korea

*** Corresponding author**

Dr. Min-Jung Kang

Center for Advanced Biomolecular Recognition

Korea Institute of Science and Technology

Seoul, 02792, Republic of Korea

Tel: +82-2-958-5088

E-mail address: [mjkang1@kist.re.kr](mailto:mjkang1@kist.re.kr)

**Fig. S3 Original full blots indicating CYP11A1, Vimentin, Snail and GAPDH for representative Western blots are used in Fig. 1A of the manuscript**

**
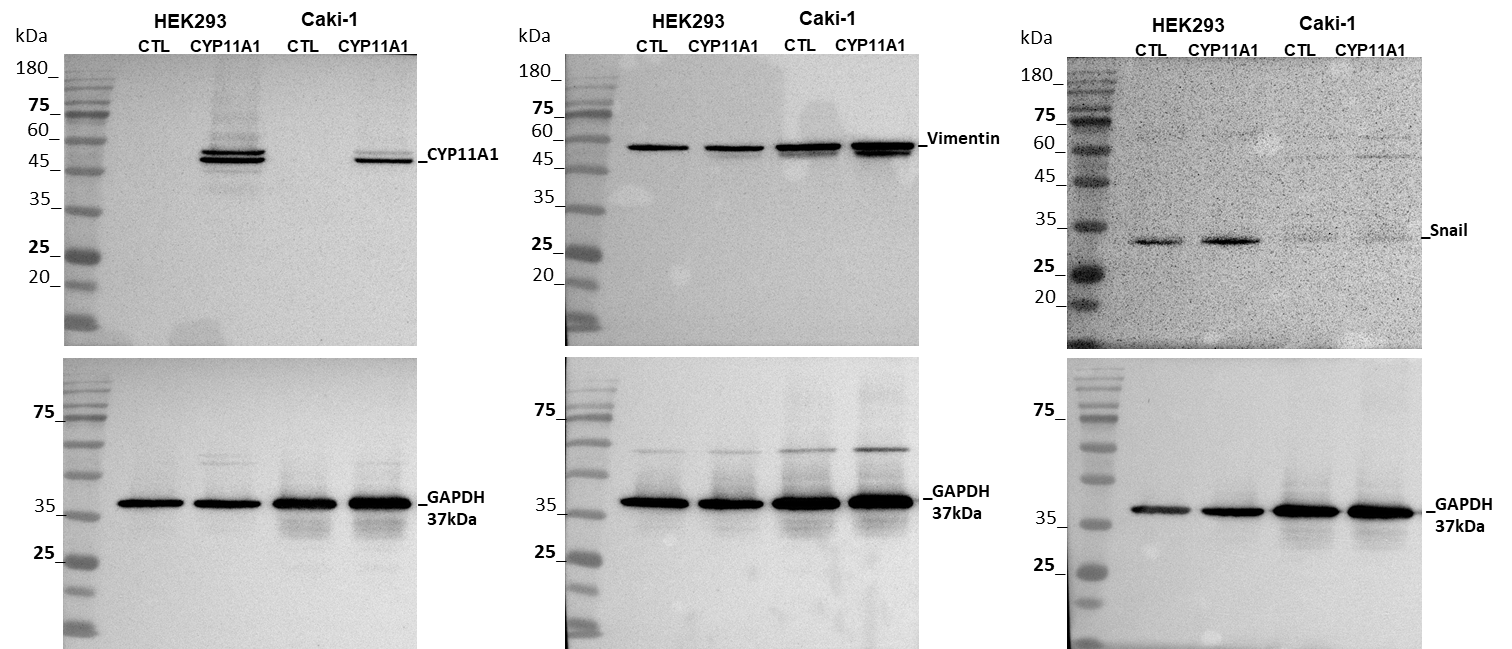
**

**Fig. S4 Original full blots indicating CylinB1, CDK2, CDK4, cdc25c, p-cdc25c, cdc2 and GAPDH for representative Western blots are used in Fig. 2D of the manuscript**

**
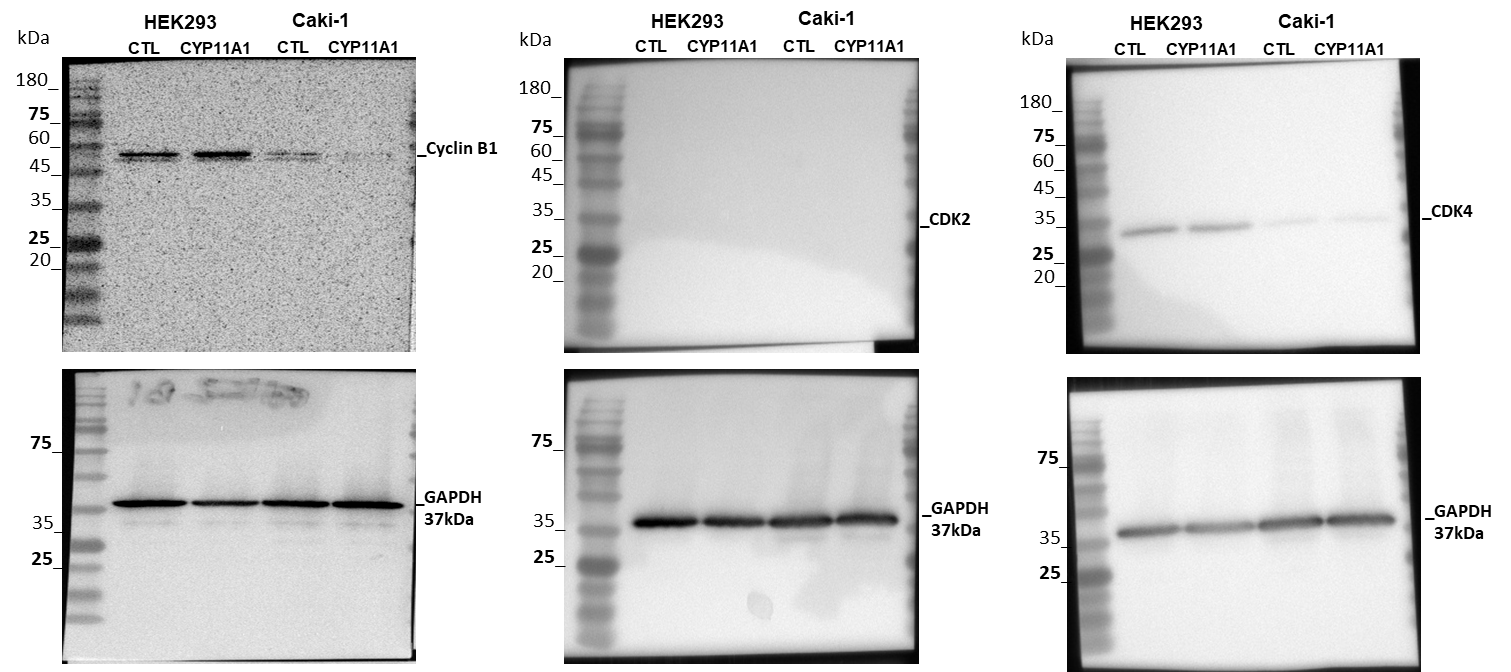
**

**
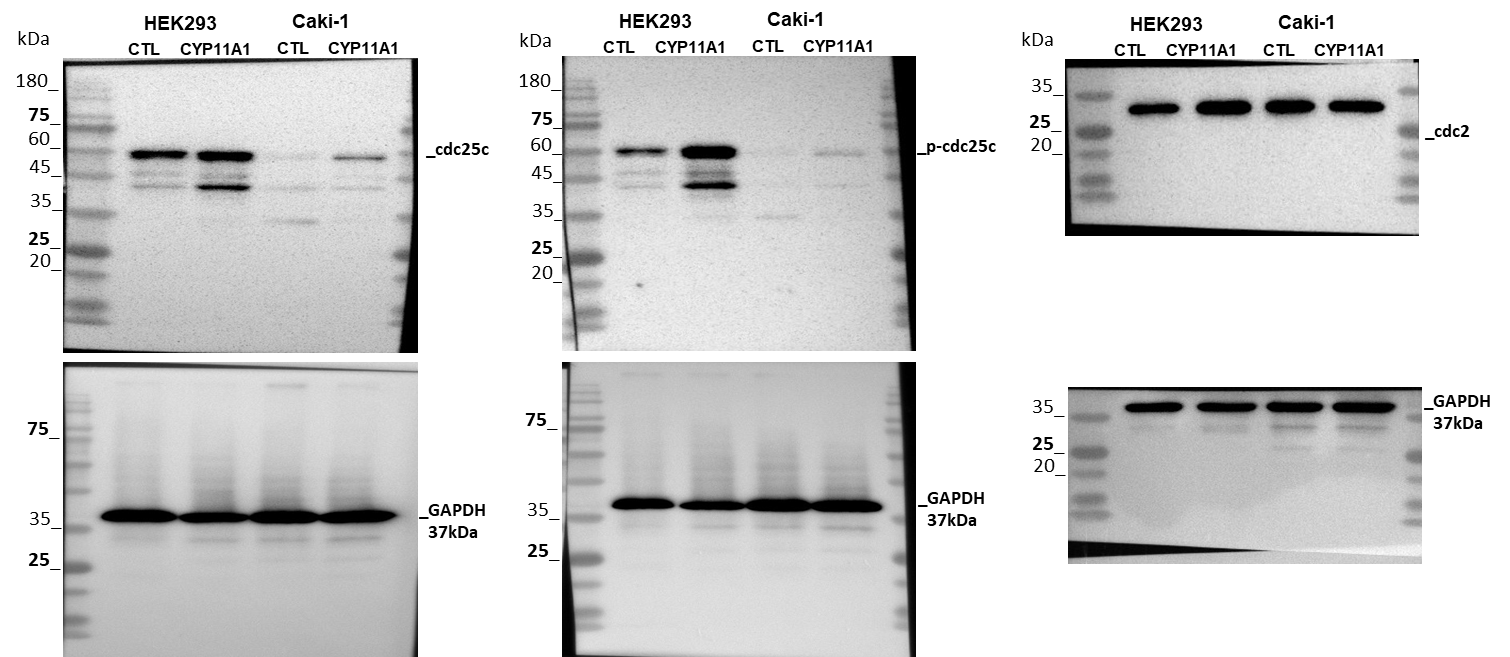
**

**Fig. S5 Original full blots indicating p-C-Raf, ERK, p-ERK, JNK, p38 and GAPDH for representative Western blots are used in Fig. 3D of the manuscript**

**
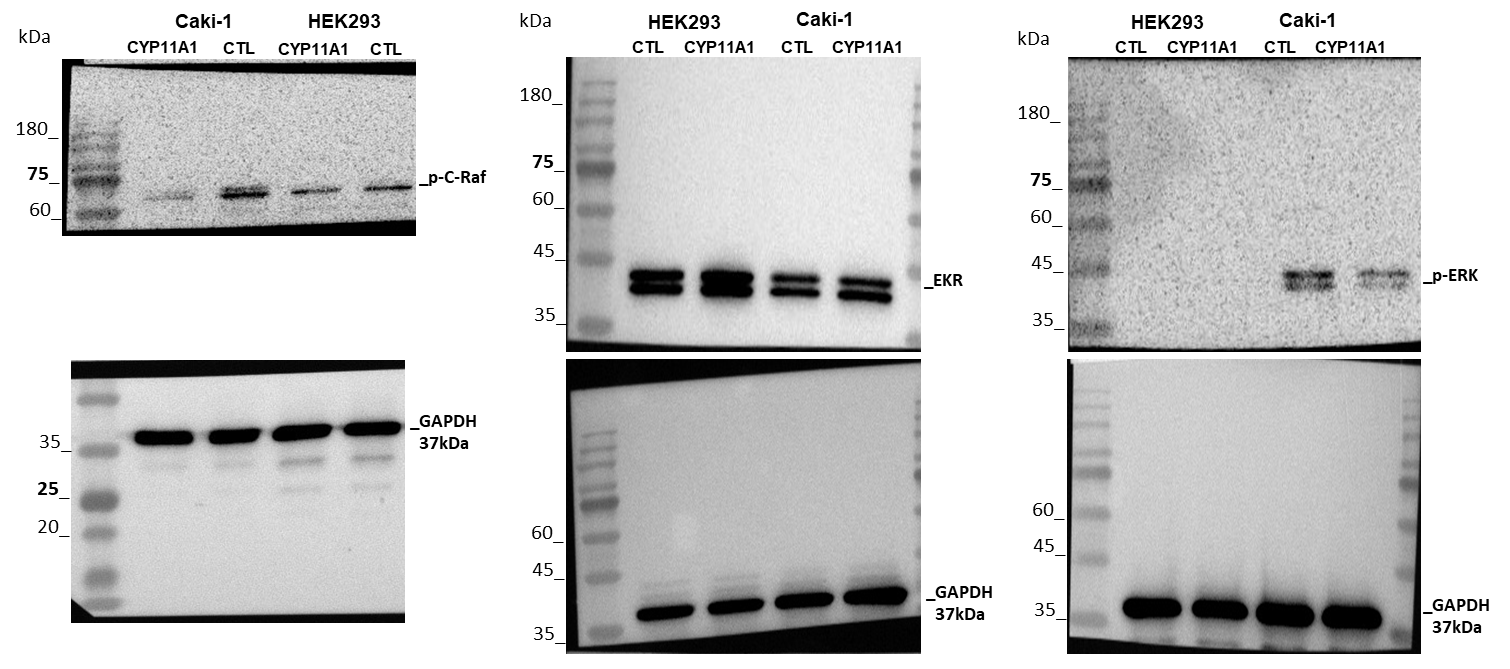
**

**
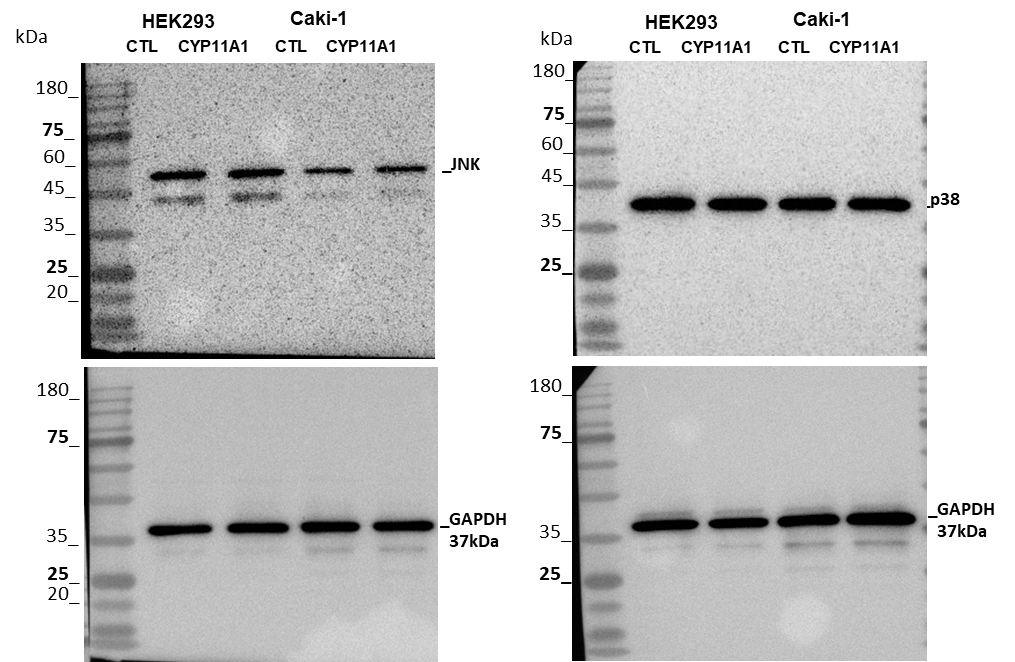
**

**Fig. S6 Original full blots indicating caspase3,7,9, PARP and GAPDH for representative Western blots are used in Fig. S2 of the Supplementary Data**

**
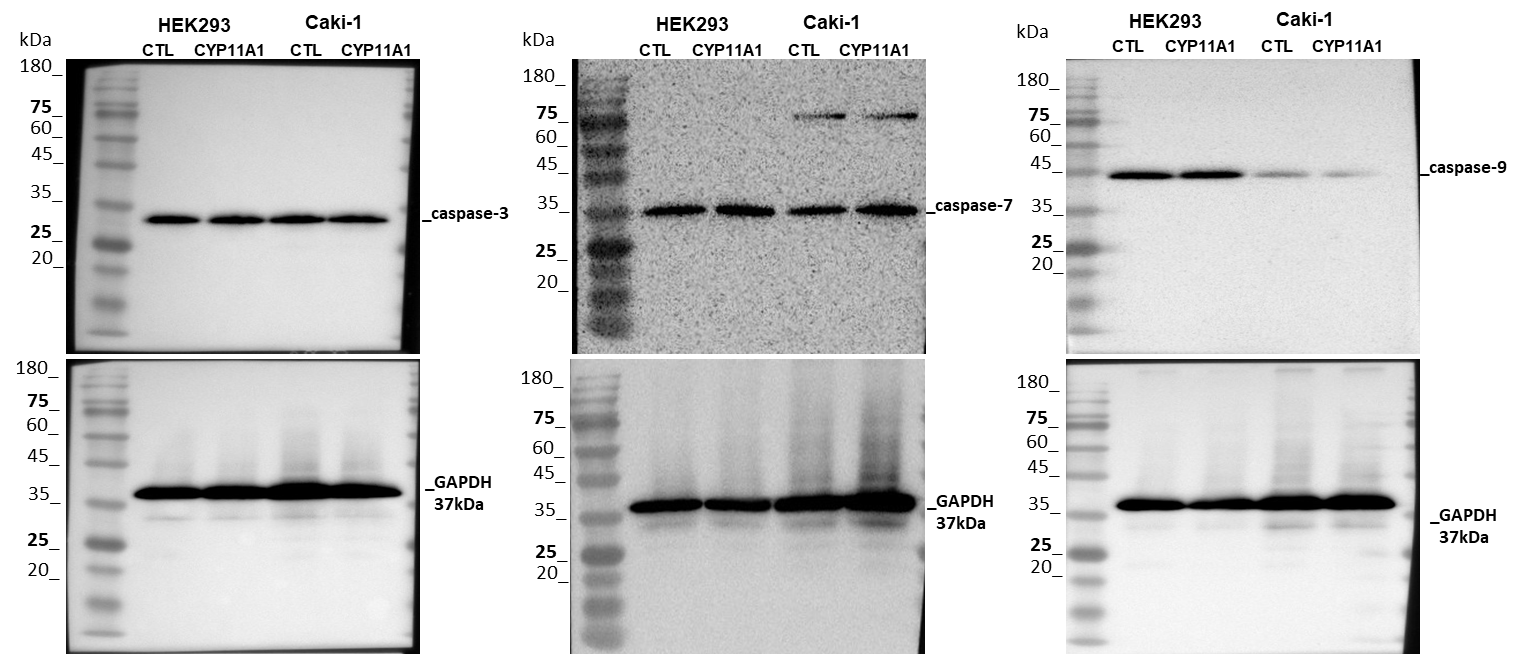
**

**
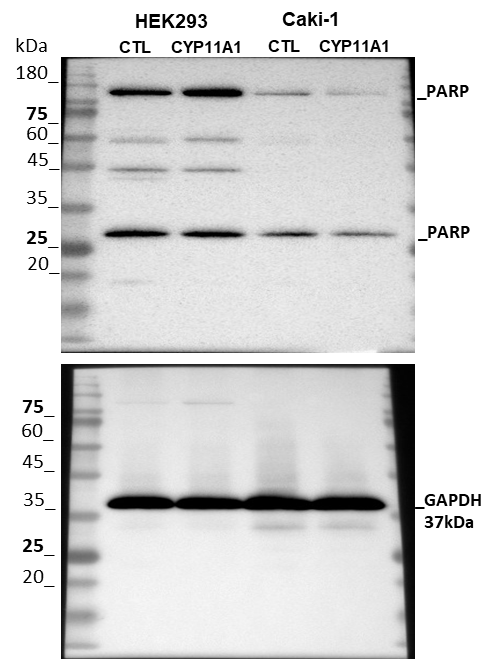
**
